# Comparative Efficacy of Interventions for Vascular Calcification and Stiffness in Dialysis Patients: A Systematic Review and Network Meta-Analysis

**DOI:** 10.1159/crm/abtag010

**Published:** 2026-09-08

**Authors:** Yitao Li, Jun Wan, Yuanyou Tang, Juan Cao, Qi Peng

**Affiliations:** a Clinical Medical College, Chengdu University, Chengdu, China; b The Second Department of Internal Medicine, Mianzhu Anorectal Specialist Hospital (Mianzhu Ziyan Street Health Service Center), Mianzhu, China

**Keywords:** Vascular calcification, Vascular stiffness, Renal dialysis, Network meta-analysis

## Abstract

**Introduction:**

Vascular calcification (VC) and vascular stiffness (VS) significantly increase cardiovascular risks in renal dialysis (RD) patients. Currently, several interventions have been tried to alleviate VC and VS, but they all lack systematic comparison. This study integrated randomized controlled trials (RCTs) of seven interventions to provide evidence-based insights to inform clinical decision-making.

**Methods:**

A systematic search was conducted for all RCTs on drug treatments for VS or VC in patients receiving RD published between January 2001 and October 2025 in four databases: PubMed, Web of Science, Embase, and The Cochrane Library. Analyses were conducted in accordance with the PRISMA network meta-analysis (NMA) guidelines and registered in PROSPERO (CRD420251060534). Risk assessment was performed using the Cochrane Risk of Bias 2.0 tool. Bayesian NMA was then conducted using R software and Stata 15.0.

**Results:**

Sodium thiosulfate (STS) showed the highest Surface Under the Cumulative Ranking Curve (SUCRA) ranking for improving pulse wave velocity (PWV) (SUCRA = 89.4%), but no statistically significant difference was observed between STS and standard care (mean difference [MD] −1.3, 95% credible interval [95% CrI] −2.8, 0.2). No statistically significant differences were observed across all interventions for PWV improvement. Etidronic acid (EA) ranked first in SUCRA for reducing coronary artery calcification (CAC) volume score (SUCRA = 84.57%), but the difference versus most interventions did not reach statistical significance (MD −1,300, 95% CrI −3,800, 1,100). STS showed statistically significant reduction in CAC volume score compared with standard care, 300 mg SNF472, and 600 mg SNF472 (MD −188.27, 95% CrI −221.68, −154.52; MD −192.55, 95% CrI −245.83, −139.54; MD −182.39, 95% CrI −237.91, −126.55). For coronary artery and aortic Agatston scores, standard care showed superior SUCRA rankings, but EA exhibited a statistically significant difference compared with cinacalcet (aortic Agatston score: MD −1,175.11, 95% CrI −2,220.92, −125.45), indicating partial comparative efficacy among drugs.

**Conclusion:**

This study suggests that SUCRA rankings indicate STS has a potential comprehensive advantage in improving VS and delaying calcification progression, making it a reasonable exploratory choice for dialysis patients with concurrent VC and VS. While EA ranks highest in SUCRA for reducing CAC volume score, it has limited impact on VS and requires caution regarding long-term safety. Given the limited sample size, network sparsity for some outcomes, and mostly non-significant between-group comparisons, large-scale, head-to-head RCTs are needed to further validate these findings.

## Introduction

End-stage kidney disease patients often require peritoneal dialysis (PD) or hemodialysis (HD) for maintenance treatment. However, vascular calcification (VC) and vascular stiffness (VS) are highly prevalent in dialysis patients and are significant risk factors for cardiovascular events and mortality [1–3].

VC is closely related to aging, chronic diseases (such as hypertension, diabetes), and metabolic disorders (such as hypercalcemia, hyperphosphatemia). This can lead to decreased vascular elasticity, lumen narrowing, and impaired blood flow, which ultimately may cause cardiovascular diseases, such as myocardial infarction and stroke. In severe cases, these conditions may threaten life. Accordingly, delaying or reversing the progression of VC and VS is of critical clinical significance for improving the cardiovascular prognosis of dialysis patients.

Existing research shows that VC and VS often coexist, with a mutually progressive relationship. Calcification is a core driver of stiffness, but stiffness may appear early in the calcification process and accelerate it [4]. The underlying mechanisms are complex, involving mineral metabolism disorders, reduced inhibitors of mineralization [5–7], inflammation of vascular elastic fibers, and high blood pressure, which cause initial vascular wall calcification or damage, leading to mild stiffness. As calcium-phosphate deposition progresses, elastic fibers are progressively destroyed, worsening VS. This damage makes the vessel wall more susceptible to further calcium-phosphate deposition, thus further exacerbating both calcification and stiffness.

### Rationale for Selected Interventions

Seven pharmacological interventions were included in this analysis based on clinical relevance and available RCT evidence: Sodium thiosulfate (STS): A reducing agent with calcium-chelating and antioxidant properties, widely investigated for anti-calcification effects in dialysis patients [8–11]. Etidronic acid (EA): A bisphosphonate that binds to calcium crystals and inhibits mineral aggregation, with reported effects on aortic calcification in HD populations [12]. Cinacalcet: A calcimimetic agent that reduces parathyroid hormone (PTH) levels, targeting secondary hyperparathyroidism as a key driver of VS [13, 14]. SNF472: An intravenous myoinositol hexaphosphate formulation that inhibits hydroxyapatite crystal growth, developed specifically for cardiovascular calcification in dialysis patients [15]. Vitamin K1 (VK1) and vitamin K2 (VK2; menaquinone-7): Regulators of matrix Gla protein activation, a key endogenous inhibitor of VC [16–18]. Atorvastatin: A statin with pleiotropic vascular effects, proposed to delay arterial stiffness progression in chronic kidney disease (CKD) [19].

All interventions have been evaluated in RCTs for VC or VS outcomes in dialysis populations and represent the main pharmacotherapeutic options investigated in this field. Previous studies have mostly focused on single-drug interventions with small sample sizes and limited follow-up, yielding fragmented findings and inconsistent conclusions. Traditional pairwise meta-analyses can only compare two interventions at a time and cannot address the scarcity of head-to-head trials across multiple agents or generate comprehensive efficacy rankings. Network meta-analysis (NMA) enables indirect comparison of multiple interventions and probabilistic ranking, which is critical for clinical decision-making when direct evidence is insufficient. This study therefore performed a Bayesian NMA to compare the relative efficacy and safety of seven pharmacological agents for improving VC and VS in dialysis patients, aiming to identify promising treatment strategies and inform clinical practice and future research.

## Methods

### Registration and Protocol

This NMA was conducted according to the Preferred Reporting Items for Systematic Reviews and Meta-Analyses (PRISMA) guidelines. Since only aggregated data were analyzed, informed consent from patients and ethical approval from an ethics committee were not required. Our study has been registered in PROSPERO with registration number ID: CRD420251060534.

### Search Strategy and Literature Source

Two researchers (LYT and TYY) independently searched four databases – PubMed, Web of Science, Embase, and The Cochrane Library – using search terms related to VC, VS, PD, HD, and RCTs from January 2001 to October 2025 (updated search). The search strategy included Medical Subject Headings terms and corresponding free-text words, combined with Boolean operators “OR” and “AND.” The detailed search strategy is provided in the online supplementary file 1 (for all online suppl. material, see https://doi.org/10.1159/crm/abtag010). Additionally, the reference lists of the retrieved studies were reviewed to ensure the inclusion of all relevant studies. To ensure the comprehensiveness of the search and minimize potential publication bias, we additionally searched the ClinicalTrials.gov registry to identify ongoing or completed but unpublished trials. Non-English studies were also excluded to minimize bias from inconsistent data interpretation and ensure the accuracy of evidence synthesis. The specific search process is detailed in online supplementary documents: Document retrieval. This systematic review was performed in accordance with the PRISMA statement. The completed PRISMA checklist with line-number cross-references to this manuscript is provided as online supplementary material.

### Inclusion Criteria for Study Selection

Studies that met the following inclusion criteria were included in the analysis: (1) Participants: Adult patients (≥18 years) diagnosed with end-stage renal disease or those undergoing regular HD or PD treatment for more than 3 months, and who met the indications for renal dialysis. (2) Interventions: Patients in the experimental group received a specific dose or treatment course of STS (All included studies using STS adopted intravenous administration; oral STS was not included due to limited clinical data), cinacalcet, SNF472, EA, menaquinone-7, VK1, or atorvastatin. (3) Control measures: Patients in the control group received standard care, saline, or placebo, with other conditions consistent between experimental and control groups. (4) Outcome measures: The study must report at least one of the following outcomes: coronary artery calcification volume score (CAC), Agatston score (differentiating aortic and coronary arteries), aortic valve calcium score (AVC), or pulse wave velocity (PWV). Each outcome measure must include baseline values or differences before and after treatment. (5) Study type: RCTs.

Studies were excluded if they met at least one of the following criteria: (1) systematic reviews, meta-analyses, case reports, conference abstracts, animal studies, or letters to the editor; (2) studies for which the full text could not be accessed, or only abstracts were available; (3) non-English language studies; (4) duplicate publications or retracted articles; (5) studies involving CKD patients who were not on dialysis.

### Literature Screening

Two researchers (LYT and TYY) conducted the literature search strictly according to the inclusion and exclusion criteria. EndNote X9 software was used for managing all retrieved articles. After importing the searched articles into EndNote X9 and excluding duplicate publications, the researchers initially screened the studies by title or abstract. Full-text articles were then downloaded and further screened to identify studies that met the criteria for this systematic review. In case of disagreement during the screening process, a third researcher intervened, and the three researchers discussed the issue until a consensus was reached.

### Data Extraction

Two researchers (LYT and TYY) independently extracted data from the studies that met the inclusion criteria for this systematic review. After extracting the basic information from the studies, they cross-checked the data and standardized the units of measurement. In cases of discrepancies in data extraction, the two researchers first rechecked the original study. If disagreements persisted, a third researcher (with expertise in meta-analysis and nephrology) independently extracted the data and resolved them through discussion with a third reviewer until consensus was reached. The extracted information included the title, first author, publication year, country of the author, country of the study population, disease background, treatment methods (dosage, administration route, frequency, duration), sample size, patient gender, age, dialysis vintage, baseline calcification score, follow-up duration, outcome measures, trial registration number, and funding source.

### Assessment of Literature Quality

Two researchers (LYT and TYY) independently assessed the quality of the studies. The Cochrane Risk of Bias 2.0 tool was used to evaluate the risk of bias in the included RCTs [20]. The assessment covered the following five domains: (1) Randomization process; (2) deviations from intended interventions; (3) missing outcome data; (4) measurement of the outcome; (5) selection of the reported result. Each of these domains was classified into three levels: low risk, some concerns, and high risk, consistent with the RoB 2.0 framework.

For studies or domains where there was disagreement between the two researchers, they discussed and analyzed the issues to reach a consensus. If no consensus could be reached, the third researcher made the final judgment after thorough discussion to resolve any remaining disagreements and ensure a fully unified conclusion. Sensitivity analysis was performed by excluding studies with some concerns or high overall risk of bias to assess the impact of study quality on results.

### Statistical Analysis

Three core assumptions underpin NMA: homogeneity, transitivity, and consistency [21]. Homogeneity was evaluated using the *I*^2^ statistic (Higgins’ heterogeneity index). A fixed-effects model was used when *I*^2^ ≤ 50%, and a random-effects model was applied when *I*^2^ > 50% to account for between-study heterogeneity.

Transitivity was evaluated qualitatively by comparing baseline patient characteristics (age, dialysis vintage, baseline calcification burden), intervention protocols, and outcome measurement methods across included studies. The clinical comparability of included populations supported the transitivity assumption.

Consistency between direct and indirect comparisons was evaluated via node-splitting analysis and inconsistency factors. No significant inconsistency was detected in closed loops across all outcomes. We used non-informative priors (normal distribution with mean = 0 and variance = 1,000) for effect sizes in the Bayesian model. Convergence was assessed using Gelman-Rubin statistics (*R* < 1.1) and trace plots, with all models achieving satisfactory convergence. This study visually assessed the connectivity of the network for different interventions and found no evidence of disconnected networks.

According to the current PRISMA NMA guidelines, the treatment network is depicted as a graph structure, with individual interventions represented as nodes and connecting lines (edges) signifying direct comparative trials between them. Larger nodes indicate higher participant numbers per intervention, while thicker edges denote a greater volume of studies comparing the respective pairs. The effect estimates are presented as mean differences (MDs) with 95% credible intervals (CrIs). MDs were used because outcome measures (e.g., PWV, CAC volume score) were reported using consistent units across studies, eliminating the need for standardized MD. A result was not considered statistically significant if its 95% CrI included the null value. In the network plot, the connecting lines represent the direct comparisons between interventions, with the width of each line corresponding to the number of studies involved in that comparison. The size of each node indicates the total sample size for that intervention. Pairwise comparisons were illustrated using interval plots to present all pairwise comparisons across included interventions. Additionally, the Surface Under the Cumulative Ranking Curve (SUCRA) was used to rank the relative efficacy of each treatment. SUCRA values range from 0 to 1 (or 0%–100% when expressed as percentages), with higher values indicating better treatment efficacy. SUCRA values were derived from the cumulative ranking probabilities based on MCMC simulations. Notably, SUCRA rankings represent probabilistic ordering only and do not equate to statistically significant superiority when 95% CrIs include the null value. To quantify uncertainty, rank probability matrices were generated to show the probability of each intervention being ranked at each possible position.

Finally, to assess potential bias due to small-scale studies in NMA, a funnel plot was drawn and visually inspected for symmetry [22]. For outcomes with fewer than 10 included studies, these tests have limited statistical power, and publication bias cannot be reliably excluded. Although no marked asymmetry was visually observed in funnel plots and Egger’s tests showed no significant asymmetry (all *p* > 0.05), these results should be interpreted with caution due to limited study numbers.

A sensitivity analysis was conducted to test the robustness of the results by excluding trials assessed as having some concerns or high overall risk of bias based on the Cochrane Risk of Bias 2.0 assessment. The Bayesian NMA was rerun including only studies judged to be at low risk of bias, and changes in effect sizes and CrI were examined. Consistent findings across analyses were interpreted as evidence of methodological robustness.

## Results

### Literature Search Process

A total of 1,833 relevant articles were retrieved. Of these, 260 articles were found in PubMed, 516 in Web of Science, 705 in Embase, and 352 in The Cochrane Library. After excluding 719 duplicate records, the remaining studies were screened by reading the titles and abstracts. Articles that did not meet the inclusion criteria, such as reviews, comments, nonclinical trials, and non-English studies, were excluded. After reviewing the full texts of the initially selected studies, 11 studies were excluded for not meeting the intervention criteria, 8 studies were excluded due to inability to access the full text, 13 studies did not meet the participant criteria, and 3 studies were not RCTs. Ultimately, 12 studies were included in the analysis. The flowchart and results of the literature search are shown in Figure 1.

**Fig. 1.**
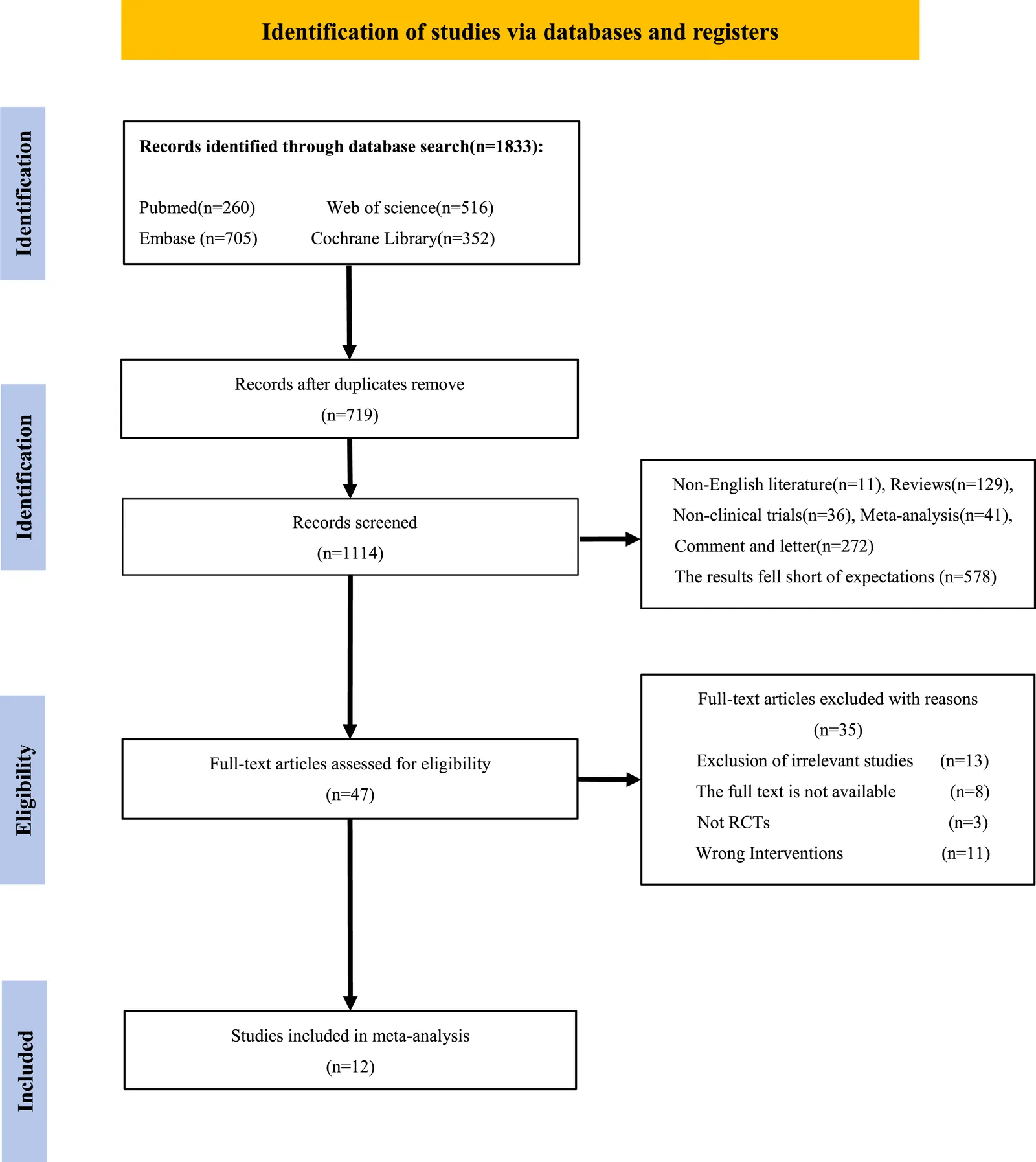
PRISMA flow chart of included studies.

### Basic Characteristics of Included Studies

The studies included were published between 2006 and 2023, covering 10 countries in Asia, Europe, and North Africa, with a majority of the studies conducted in Asia. All studies were parallel-controlled, prospective RCTs, and the participants were end-stage renal disease patients undergoing PD or HD for ≥3 months. The average age of participants was around 60 years, with a slightly higher proportion of males (approximately 59%). The follow-up duration of most studies was 6–12 months. Overall, the sample size of the studies ranged from 12 to 273 participants. The primary interventions included STS, cinacalcet, atorvastatin, VK1/K2, EA, and SNF472. The control groups received standard care or routine nursing, and some studies used saline or placebo as a control. The dialysis method, baseline treatments, and nutritional support were consistent across the trials, minimizing systematic differences between interventions. The VC indices included: CAC volume score, Agatston score (for coronary and aortic regions), and AVC score. The VS indices included PWV and Augmentation Index. Six studies reported PWV, six reported CAC volume score, six reported Agatston scores (coronary and/or aortic regions), and three reported AVC. Overall, calcification-related data were more abundant, whereas stiffness data were relatively limited; nevertheless, data were clinically comparable across studies. Detailed clinical characteristics are presented in Table 1.

**Table 1.** Characteristics of studies included in this review

Study ID	Country	Study	Sample size			Ages			Gender			Intervention mode		
			group 1	group 2	group 3	group 1	group 2	group 3	group 1	group 2	group 3	group 1	group 2	group 3
Saengpanit et al. [8] (2018)	Thailand	RCT	24	26	​	50.4±9.5	54.4±10.7	​	12m/12F	16m/10F	​	STS	Standard care	​
Raggi et al. [15] (2019)	Canada	RCT	92	92	90	63.0±9.5	63.6±8.9	64.1±8.2	54m/38F	55m/36F	57m/33F	Hexasodium phytate 300 mg (SNF472)	Hexasodium phytate 600 mg (SNF472)	Standard care
Ariyoshi et al. [12] (2006)	Japan	RCT	6	6	​	63.5±8.3	66.3±5.1	​	5m/1F	5m/1F	​	EA	Standard care	​
Elsayed et al. [19] (2022)	Egypt	RCT	25	25	​	47.72±10.26	47.20±10.81	​	15m/10F	12m/13F	​	Atorvastatin	Standard care	​
Haroon et al. [23] (2023)	Singapore	RCT	89	89	​	62 (29–80)	61 (24–78)	​	54m/35F	47m/42F	​	Vitamin K2	Standard care	​
Naiyarakseree et al. [17] (2023)	Thailand	RCT	50	46	​	59.7±11.3	60.0±11.8	​	26m/24F	26m/20F	​	MK-7	Standard care	​
Oikonomaki et al. [18] (2023)	Greece	RCT	44	58	​	70.18±12.48	66.65±16.4	​	NA	NA	​	MK-7	Standard care	​
Saritas et al. [16] (2022)	Germany	RCT	17	23	​	62.5±12.3	64.4±13.3	​	11m/6F	17m/6F	​	Vitamin K1	Standard care	​
Bian et al. [24] (2022)	China	RCT	25	25	​	52.14±20.8	52.46±19.37	​	12m/13F	12m/13F	​	STS	Standard care	​
Adirekkiat et al. [9] (2010)	Thailand	RCT	16	16	​	59.6±14.4	60.4±11.5	​	11m/5F	8m/8F	​	STS	Standard care	​
Eddington et al. [13] (2021)	UK	RCT	15	21	​	45 (Average)	54 (Average)	​	11m/4F	12m/9F	​	Cinacalcet	Standard care	​
Djuric et al. [25] (2020)	Serbia	RCT	30	30	​	63.8±13.2	64.1±9.7	​	17m/13F	21m/9F	​	STS	Standard care	​

Dosage	Duration	Outcome indicators
IV, 12.5 g, twice a week for 6 months	6 months	CAC score cfPWV
NA	12 months	CAC score
		AVC score
		Agatston score (coronary artery)
400 mg/day for 24 weeks	6 months	CAC score
		Agatston score (aorta)
10 mg atorvastatin or placebo for 24 weeks	6 months	Aortic PWV
		AIx
360 mg, 3 times weekly	12 months	CAC score
		AIx
375 μg once daily for 24 weeks	6 months	cfPWV
Orally 200 μg of vitamin K2, every day for 1 year	12 months	Agatston score (aorta)
Orally thrice weekly	12 months	CAC score
		AVC score
		TAC
		MVC
0.18 g/kg, dissolved in 100 mL normal saline three times a week for 6 months	6 months	CAC score
Twenty-five percent STS, 12.5 g IV, twice a week for a period of at least 4 months	6 months	Agatston score (coronary artery)
		CAC score
NA	12 months	cfPWV
		ACA
		Coronary calcium score
		Agatston score (coronary artery, aorta)
25 g/1.73 m^2^	6 months	PWV

MK-7, menaquinone-7.

All outcome indicators are abbreviated as follows: CAC, coronary artery calcium; cfPWV, carotid-femoral pulse wave velocity; AVC, aortic valve calcium; AIx, augmentation index; TAC, thoracic aortic calcifications; MVC, mitral valve calcium score; ACA, aortic calcification area.

### Risk of Bias in Included Studies

The risk of bias for the 12 included RCTs was assessed using the Cochrane Risk of Bias 2.0 tool [20], with results visualized in Figure 2 (risk of bias summary) and Figure 3 (percentage distribution). Overall, 9 studies were judged to have low overall risk of bias, and 3 studies raised some concerns primarily related to selective reporting and unclear outcome assessment blinding. No studies were rated as high overall risk of bias. The methodological quality of the included RCTs was generally high. Sensitivity analysis excluding studies with some concerns was conducted to test result robustness.

**Fig. 2.**
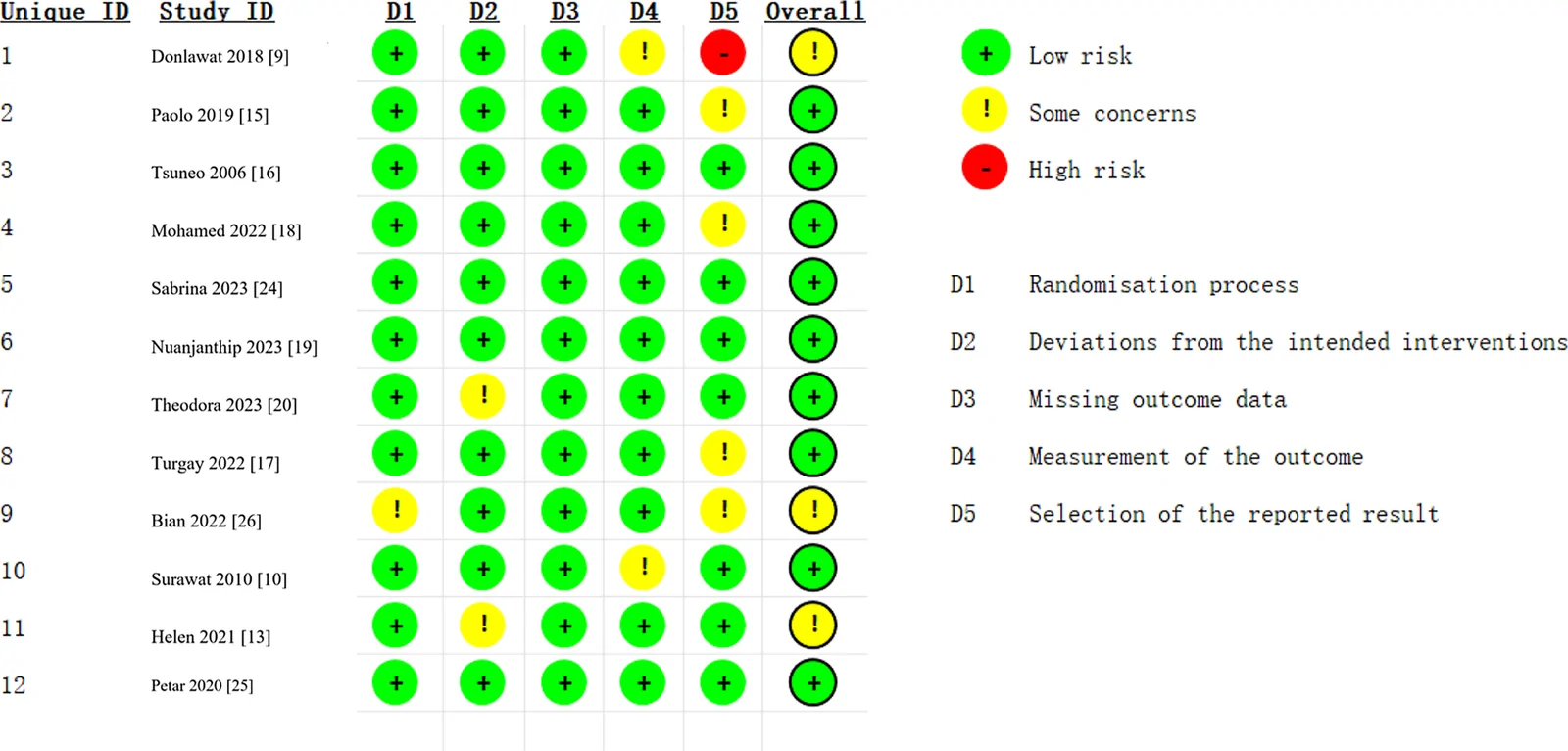
Risk of bias summary.

**Fig. 3.**
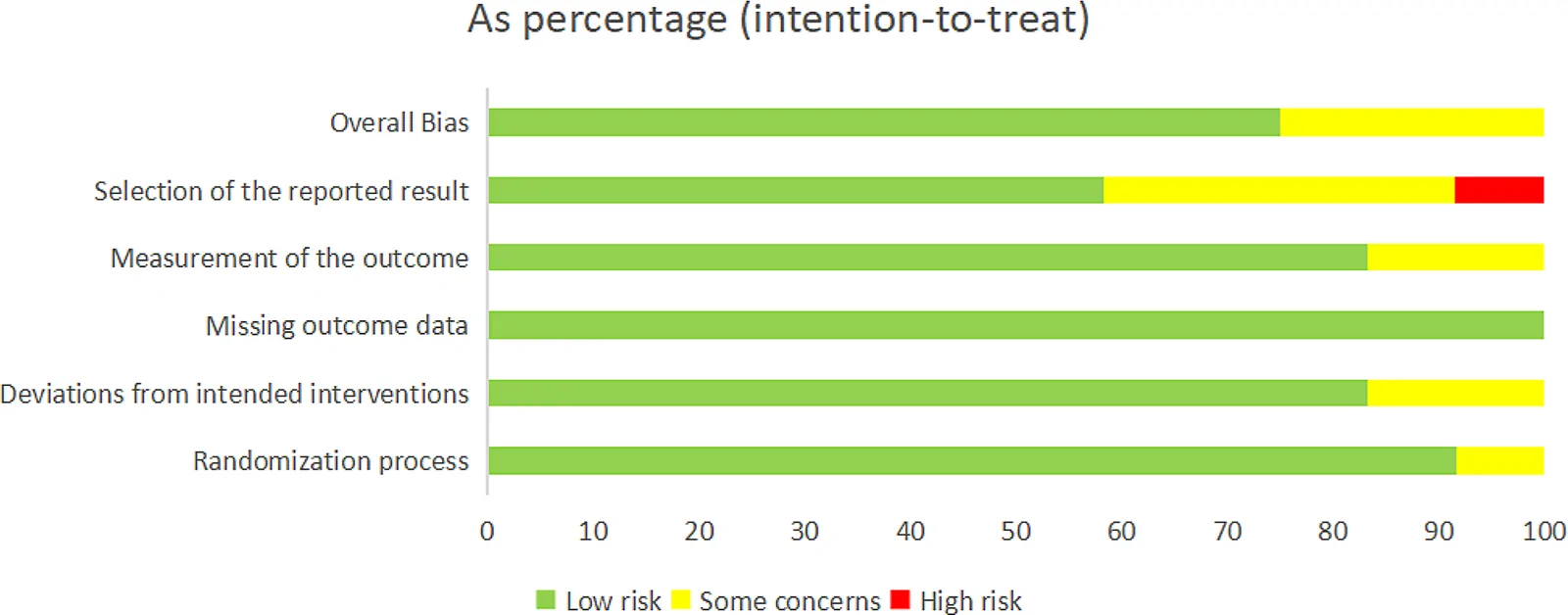
Risk of bias summary as a percentage.

### NMA Results of Included Studies

#### Summary of Network

The network plot (Fig. 4) illustrates the comparative evidence structure across seven interventions and five outcomes. Six studies used PWV to assess the effect of various drugs on VS. These studies involved four drugs: STS, cinacalcet, atorvastatin, and VK2 (Fig. 4a). Six studies used the CAC volume score to evaluate the effects of five drugs – STS, EA, SNF472, VK1, and VK2 – on VC (Fig. 4b). When the Agatston score was used to assess improvements in VC, the evaluations were categorized by the effects on coronary and aortic artery calcification. Among these, STS, cinacalcet, and SNF472 were assessed for their effects on coronary artery calcification, with three studies involved (Fig. 4c). EA, cinacalcet, and VK2 were assessed for their effects on aortic artery calcification, with three studies involved (Fig. 4d). Finally, three studies used AVC as an indicator to assess the effects of SNF472, VK1, and VK2 on VC (Fig. 4e). Larger nodes represent larger sample sizes, and thicker edges represent more head-to-head trials. Notably, networks for Agatston scores and AVC are relatively sparse due to limited included studies, and ranking results for these outcomes should be interpreted cautiously.

**Fig. 4.**
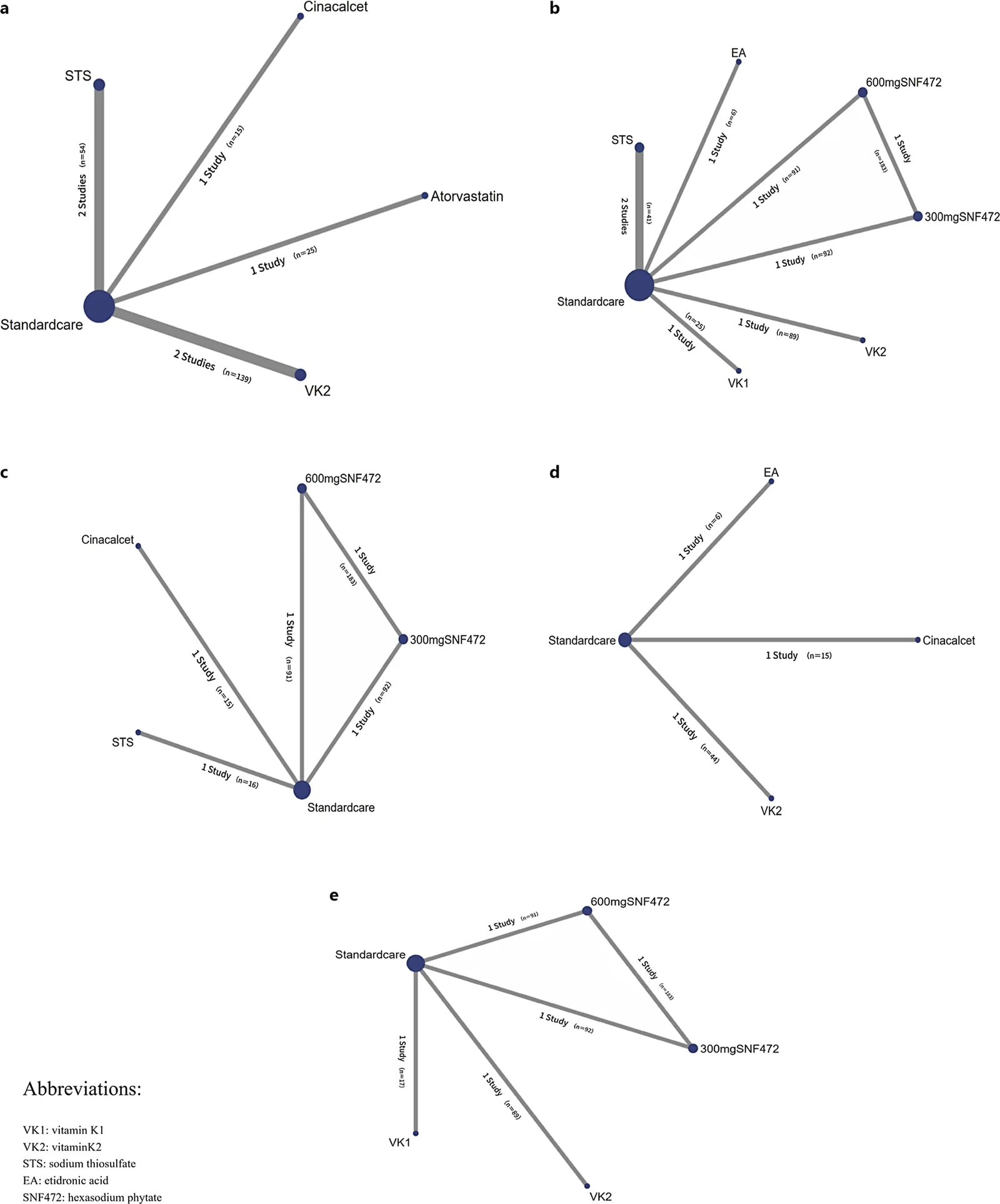
The network diagram under the evaluation of five different indicators. **a** Network diagram for pulse wave velocity (PWV), including four interventions: STS, cinacalcet, atorvastatin, VK2; (**b**) network diagram for coronary artery calcification (CAC) volume score, including five interventions: STS, EA, SNF472, VK1, VK2; (**c**) network diagram for coronary artery Agatston score, including three interventions: STS, cinacalcet, SNF472; (**d**) network diagram for aortic Agatston score, including three interventions: EA, cinacalcet, VK2; (**e**) network diagram for aortic valve calcification (AVC), including three interventions: SNF472, VK1, VK2. *Note: STS, sodium thiosulfate; EA, etidronic acid; VK1, vitamin K1; VK2, vitamin K2; SNF472, intravenous myoinositol hexaphosphate.

#### Effect of Different Interventions on PWV

A total of six studies included 233 dialysis patients and provided corresponding PWV data [8, 13, 17, 19, 23, 25]. Bayesian NMA results indicated that there were no statistically significant differences between any pair of interventions (Table 2). SUCRA rankings indicated that STS had the greatest numerical advantage for arterial stiffness improvement (SUCRA = 89.4%) (Fig. 5). However, since no pairwise comparison reached statistical significance, this ranking only reflects a numerical trend and does not confirm definitive superiority of STS over other interventions. SUCRA values represent point estimates; the uncertainty of ranking distributions is further visualized in online supplementary Figures S1–S5 (rank probability stacked bar charts).

**Table 2.** League table of efficacy of different drugs for improving PWV

Atorvastatin	**0.08 (−2.93, 3.13)**	**0.19 (−1.88, 2.26)**	**−1.09 (−3.66, 1.43)**	**0.49 (−1.96, 2.97)**
−0.08 (−3.13, 2.93)	Cinacalcet	0.11 (−2.1, 2.31)	−1.16 (−3.88, 1.47)	0.42 (−2.17, 2.99)
−0.19 (−2.26, 1.88)	−0.11 (−2.31, 2.1)	Standard care	−1.28 (−2.8, 0.2)	0.3 (−1.05, 1.66)
1.09 (−1.43, 3.66)	1.16 (−1.47, 3.88)	1.28 (−0.2, 2.8)	STS	1.58 (−0.42, 3.64)
−0.49 (−2.97, 1.96)	−0.42 (−2.99, 2.17)	−0.3 (−1.66, 1.05)	−1.58 (−3.64, 0.42)	VK2

Values are presented as MD (95% CrI). Bold values indicate statistically significant results (95% credible interval does not include null value). Values are calculated as row intervention minus column intervention. Negative MD values indicate lower vascular calcification or stiffness (better efficacy) for the row intervention.

STS, sodium thiosulfate; VK2, vitamin K2.

**Fig. 5.**
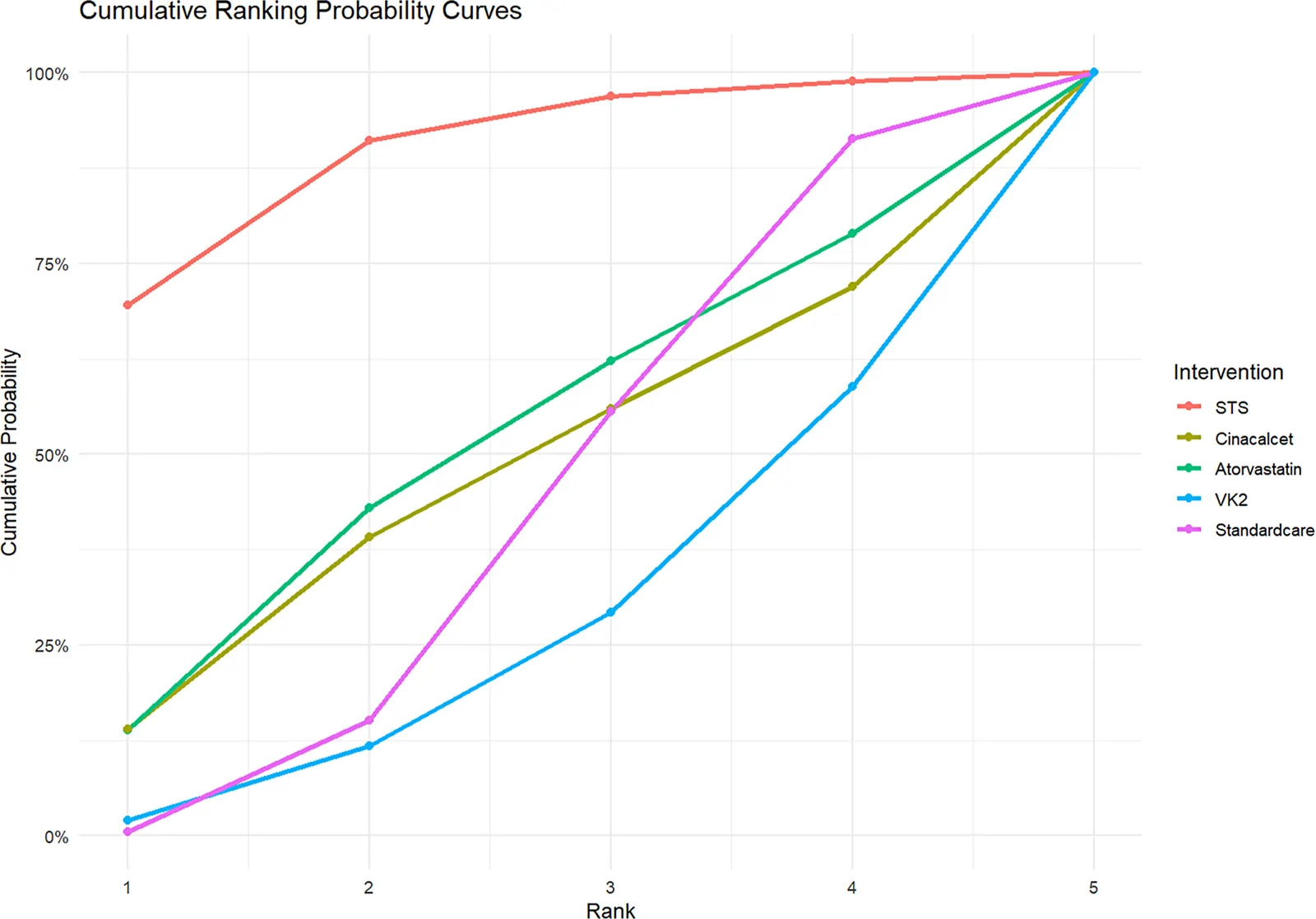
Cumulative ranking probability curves for pulse wave velocity (PWV) improvement. *Note: STS, sodium thiosulfate; VK2, vitamin K2. SUCRA values represent probabilistic ranking only.

#### Effect of Different Interventions on the CAC Volume Score

This study also analyzed the CAC volume score to explore the effect of the included drugs on VC. Six trials reported CAC volume scores for 245 dialysis patients [9, 12, 15, 16, 23, 24]. Bayesian NMA results indicated that STS showed statistically significant differences compared to standard care (MD −188.27, 95% CrI −221.68, −154.52), 300 mg SNF472 (MD −192.55, 95% CrI −245.83, −139.54), and 600 mg SNF472 (MD −182.39, 95% CrI −237.91, −126.55). VK2, when compared to STS (MD 574.39, 95% CrI 346.36, 800.34), standard care (MD 386.58, 95% CrI 161.18, 609.66), 300 mg SNF472 (MD 382.12, 95% CrI 152.9, 609.67), and 600 mg SNF472 (MD 392.39, 95% CrI 162.04, 620.23), also showed statistically significant differences (Table 3).

**Table 3.** League table of efficacy of different drugs for improving CAC volume score

300 mg SNF472	**−10.34 (−61.46, 40.9)**	**−1,334.62 (−3,815.97, 1,142.72)**	**−4.44 (−46.07, 37.11)**	**−192.55 (−245.83, −139.54)**	**−271.66 (−1,157.12, 617.76)**	**382.12 (152.9, 609.67)**
10.34 (−40.9, 61.46)	600 mg SNF472	−1,323.46 (−3,804.01, 1,150.88)	5.89 (−38.74, 50.46)	**−182.39 (−237.91, −126.55)**	−261.34 (−1,147.35, 627.91)	**392.39 (162.04, 620.23)**
1,334.62 (−1,142.72, 3,815.97)	1,323.46 (−1,150.88, 3,804.01)	EA	1,330.06 (−1,145.84, 3,810.76)	1,142.33 (−1,333.71, 3,621.79)	1,064.31 (−1,570.91, 3,690.65)	1,717.81 (−769.14, 4,202.51)
4.44 (−37.11, 46.07)	−5.89 (−50.46, 38.74)	−1,330.06 (−3,810.76, 1,145.84)	Standard care	**−188.27 (−221.68, −154.52)**	−267.03 (−1,151.02, 622.05)	**386.58 (161.18, 609.66)**
**192.55 (139.54, 245.83)**	**182.39 (126.55, 237.91)**	−1,142.33 (−3,621.79, 1,333.71)	**188.27 (154.52, 221.68)**	STS	−79.22 (−963.13, 810.81)	**574.39 (346.36, 800.34)**
271.66 (−617.76, 1,157.12)	261.34 (−627.91, 1,147.35)	−1,064.31 (−3,690.65, 1,570.91)	267.03 (−622.05, 1,151.02)	79.22 (−810.81, 963.13)	VK1	653.52 (−261.22, 1,568.93)
**−382.12 (−609.67, −152.9)**	**−392.39 (−620.23, −162.04)**	−1,717.81 (−4,202.51, 769.14)	**−386.58 (−609.66, −161.18)**	**−574.39 (−800.34, −346.36)**	−653.52 (−1,568.93, 261.22)	VK2

Values are presented as MD (95% CrI). Bold values indicate statistically significant results (95% credible interval does not include null value). Values are calculated as row intervention minus column intervention. Negative MD values indicate lower vascular calcification or stiffness (better efficacy) for the row intervention.

EA, etidronic acid; STS, sodium thiosulfate; VK1, vitamin K1; VK2, vitamin K2; SNF472, intravenous myoinositol hexaphosphate.

Notably, the SUCRA ranking indicated that EA ranked first with a SUCRA value of 84.57%, followed by STS (SUCRA = 76.9%) and VK1 (SUCRA = 64.5%) (Fig. 6). However, EA did not show statistically significant differences versus most comparators, and its top ranking should be interpreted as a probabilistic trend rather than confirmed superior efficacy.

**Fig. 6.**
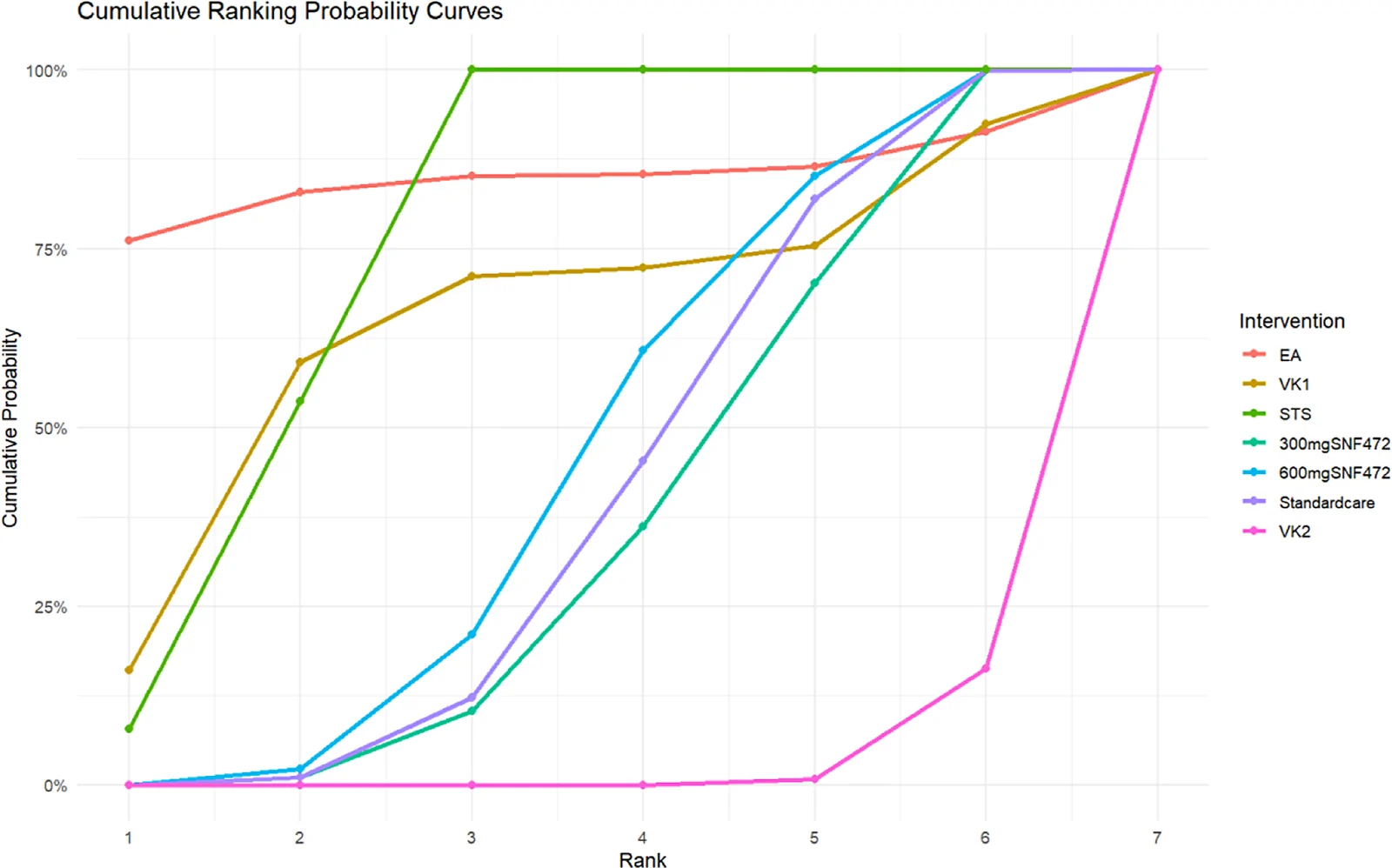
Cumulative ranking probability curves for CAC volume score improvement. *Note: EA, etidronic acid; STS, sodium thiosulfate; SNF472, intravenous myoinositol hexaphosphate; VK1, vitamin K1; VK2, vitamin K2. SUCRA values represent probabilistic ranking only.

#### Effect of Different Interventions on Coronary Artery Agatston Score

Three studies reported the coronary artery Agatston scores for 123 dialysis patients [9, 13, 15]. Bayesian NMA of the results for the four included drugs showed no statistically significant differences between the drugs (Table 4). Standard care achieved the highest SUCRA ranking (SUCRA = 76.1%) for coronary artery calcification reduction (Fig. 7). Given the small number of included studies and sparse network structure, this ranking result has limited stability and should be interpreted with caution.

**Table 4.** League table of efficacy of different drugs for improving coronary artery Agatston score

300 mg SNF472	**20.69 (−188.38, 228.52)**	**−99.2 (−396.54, 195.31)**	**64.7 (−144.63, 270.96)**	**−16.15 (−1,691.51, 1,687.3)**
−20.69 (−228.52, 188.38)	600 mg SNF472	−119.86 (−415.06, 175.55)	43.89 (−165.32, 250.17)	−37.15 (−1,711.05, 1,667.13)
99.2 (−195.31, 396.54)	119.86 (−175.55, 415.06)	Cinacalcet	163.76 (−45.91, 374.81)	83.69 (−1,590, 1,785.2)
−64.7 (−270.96, 144.63)	−43.89 (−250.17, 165.32)	−163.76 (−374.81, 45.91)	Standard care	−80.18 (−1,743.04, 1,610.37)
16.15 (−1,687.3, 1,691.51)	37.15 (−1,667.13, 1,711.05)	−83.69 (−1,785.2, 1,590)	80.18 (−1,610.37, 1,743.04)	STS

Values are presented as MD (95% CrI). Bold values indicate statistically significant results (95% credible interval does not include the null value). Values are calculated as row intervention minus column intervention. Negative MD values indicate lower vascular calcification or stiffness (better efficacy) for the row intervention.

STS, sodium thiosulfate; SNF472, intravenous myoinositol hexaphosphate.

**Fig. 7.**
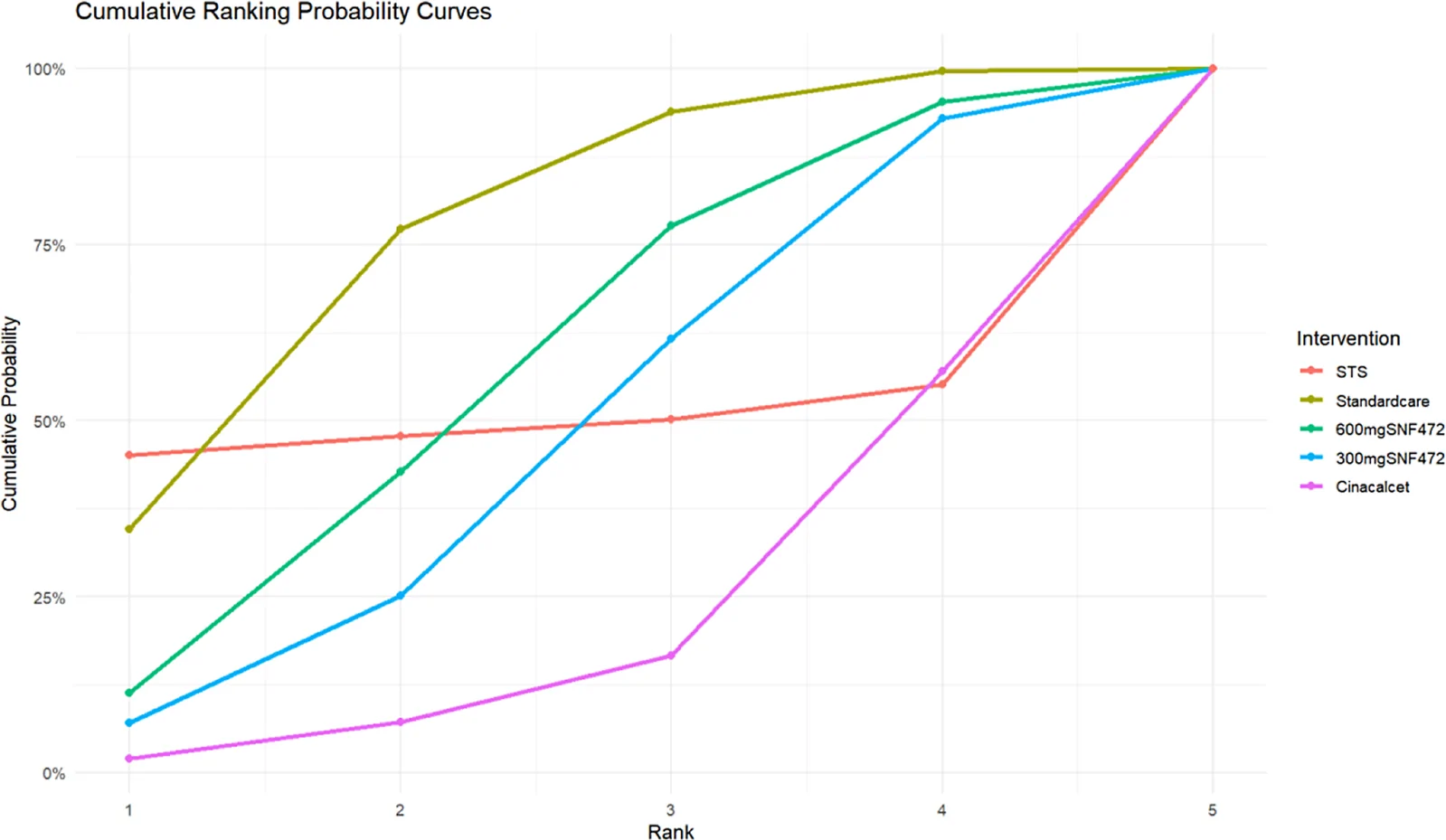
Cumulative ranking probability curves for coronary artery Agatston score improvement. * Note: STS, sodium thiosulfate; SNF472, intravenous myoinositol hexaphosphate. SUCRA values represent probabilistic ranking only.

#### Effect of Different Interventions on Aortic Agatston Score

Three studies reported the aortic Agatston scores for 65 dialysis patients [12, 13, 18]. Standard care achieved the highest SUCRA ranking (SUCRA = 84.8%) for aortic calcification reduction (Fig. 8).

**Fig. 8.**
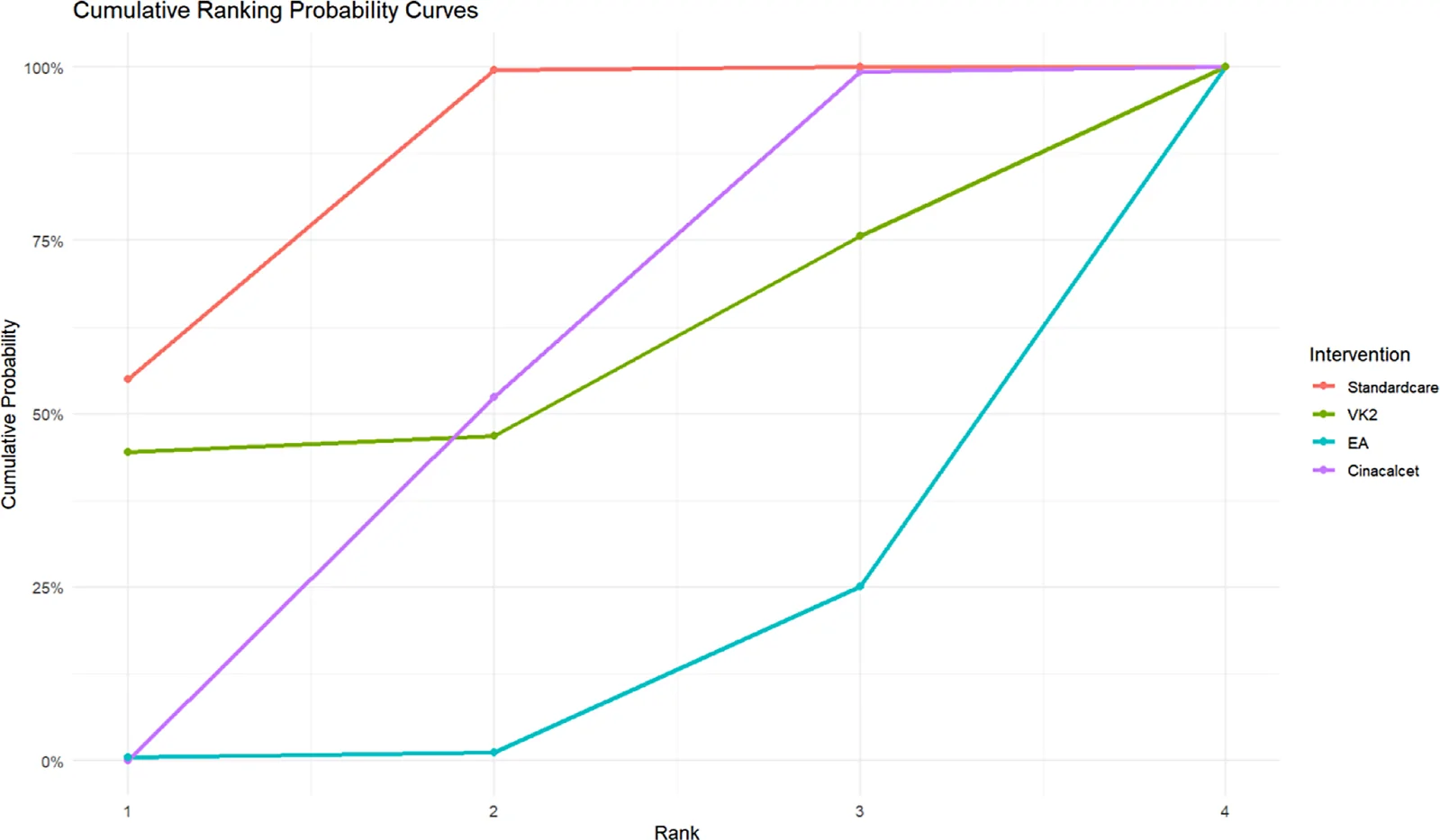
Cumulative ranking probability curves for aortic Agatston score improvement. *Note: EA, etidronic acid; VK2, vitamin K2. SUCRA values represent probabilistic ranking only.

Bayesian NMA results showed that EA had a statistically significant difference compared to cinacalcet (MD −1,175.11, 95% CrI −2,220.92, −125.45). Furthermore, standard care was associated with significantly higher aortic Agatston scores (i.e., worse calcification) compared with EA (MD 1261.55, 95% CrI 212.02–2,304.27) and cinacalcet (MD 86.03, 95% CrI 35.36, 136.5) (Table 5). This outcome is based on only 3 studies with a small total sample size, and the generalizability of findings is limited.

**Table 5.** League table of efficacy of different drugs for improving aortic Agatston score

Cinacalcet	**−1,175.11 (−2,220.92, −125.45)**	**86.03 (35.36, 136.5)**	**−105.26 (−2,910.57, 2,715.1)**
**1,175.11 (125.45, 2,220.92)**	EA	**1,261.55 (212.02, 2,304.27)**	1,070.4 (−1,927.94, 4,081.13)
**−86.03 (−136.5, −35.36)**	**−1,261.55 (−2,304.27, −212.02)**	Standard care	−190.63 (−2,992.99, 2,629.02)
105.26 (−2,715.1, 2,910.57)	−1,070.4 (−4,081.13, 1,927.94)	190.63 (−2,629.02, 2,992.99)	VK2

Values are presented as MD (95% CrI). Bold values indicate statistically significant results (95% credible interval does not include null value). Values are calculated as row intervention minus column intervention. Negative MD values indicate lower vascular calcification or stiffness (better efficacy) for the row intervention.

EA, etidronic acid; VK2, vitamin K2.

#### Effect of Different Interventions on AVC

A total of 289 dialysis patients participated in three studies reporting data on AVC [15, 16, 23]. The Bayesian NMA results showed that there were no statistically significant differences between any of the treatments in the included studies (Table 6). Standard care ranked highest in SUCRA (SUCRA = 75.8%) for aortic valve calcification reduction (Fig. 9). As with other sparse networks, this ranking reflects probabilistic ordering only and should not be interpreted as evidence of superior efficacy of standard care.

**Table 6.** League table of efficacy of different drugs for improving AVC

300 mg SNF472	**−7.66 (−38.32, 23.01)**	**7.3 (−23.5, 38.02)**	**−16.91 (−72.54, 38.43)**	**5.86 (−44.03, 55.66)**
7.66 (−23.01, 38.32)	600 mg SNF472	14.93 (−15.6, 45.65)	−9.24 (−65.02, 46.32)	13.5 (−36.57, 63.38)
−7.3 (−38.02, 23.5)	−14.93 (−45.65, 15.6)	Standard care	−24.2 (−71.25, 22.79)	−1.43 (−41.43, 38.65)
16.91 (−38.43, 72.54)	9.24 (−46.32, 65.02)	24.2 (−22.79, 71.25)	VK1	22.8 (−39.4, 84.75)
−5.86 (−55.66, 44.03)	−13.5 (−63.38, 36.57)	1.43 (−38.65, 41.43)	−22.8 (−84.75, 39.4)	VK2

Values are presented as MD (95% CrI). Bold values indicate statistically significant results (95% credible interval does not include null value). Values are calculated as row intervention minus column intervention. Negative MD values indicate lower vascular calcification or stiffness (better efficacy) for the row intervention.

VK1, vitamin K1; VK2, vitamin K2; SNF472, intravenous myoinositol hexaphosphate.

**Fig. 9.**
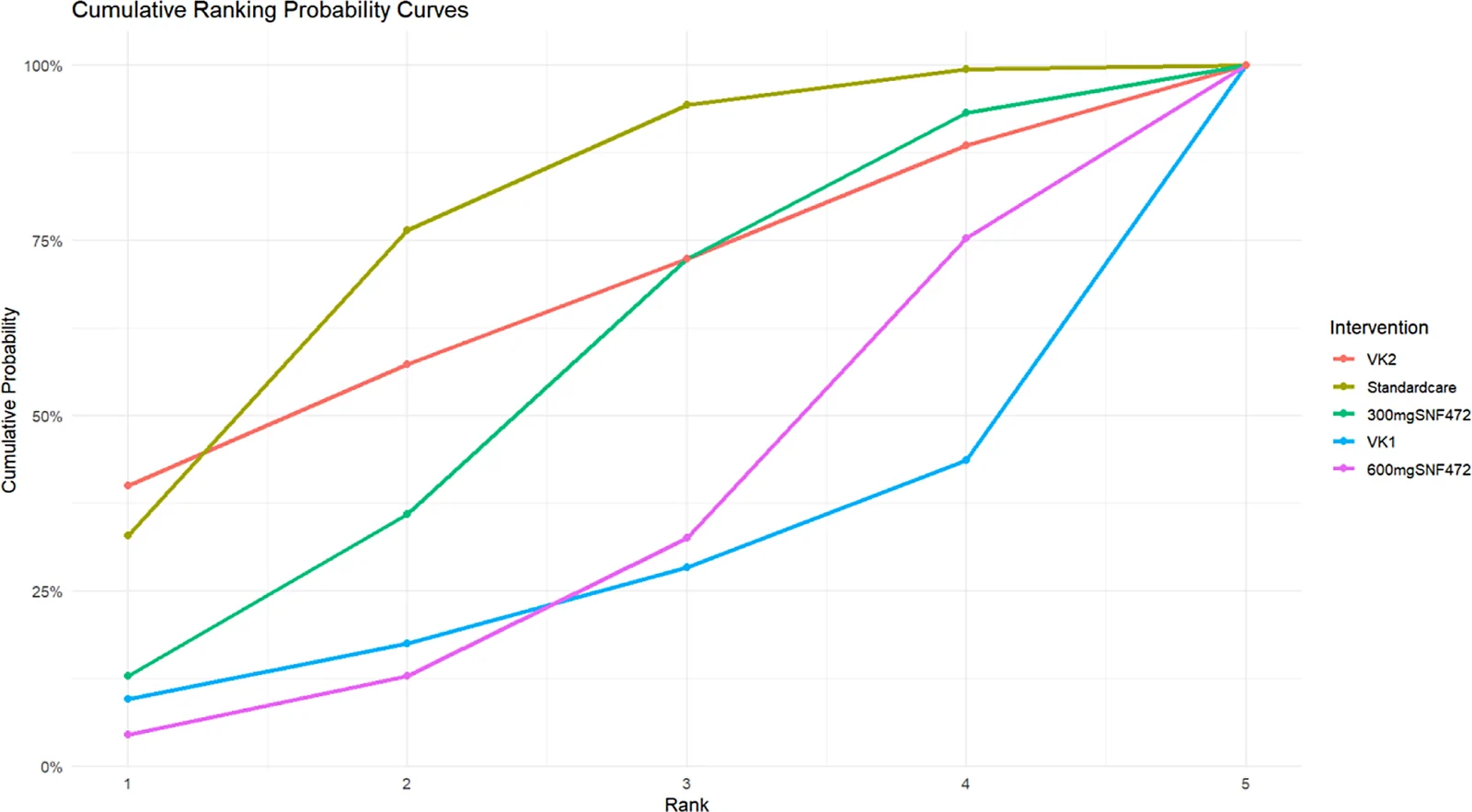
Cumulative ranking probability curves for AVC improvement. *Note: VK1, vitamin K1; VK2, vitamin K2; SNF472, intravenous myoinositol hexaphosphate. SUCRA values represent probabilistic ranking only.

### Sensitivity Analysis by Excluding Studies with Some Concerns or High Risk of Bias

To assess the robustness of our findings, we performed a sensitivity analysis limited to studies rated as having low overall risk of bias according to the Cochrane RoB 2.0 tool, with the Bayesian NMA rerun under identical analytical specifications. Results are visualized in online supplementary Figure S6A–D (four calcification endpoints) and online supplementary Figure S7A (VS endpoint), with all interventions compared against standard care.

For the two primary endpoints, the direction of effect estimates remained consistent with the primary analysis, though CrI widened substantially due to reduced sample size and fewer included studies. For pulse wave velocity (PWV; online suppl. Fig. S7A), the MD of STS versus standard care was −1.28 (95% CrI −2.80, 0.20) in the primary analysis and −1.8 (95% CrI −4.2, 0.58) in the sensitivity analysis. Neither comparison reached statistical significance, and the numerical trend favoring STS remained unchanged.

For CAC volume score (online suppl. Fig. S6D), the MD of STS versus standard care shifted from −188.27 (95% CrI −221.68, −154.52) in the primary analysis to −39 (95% CrI −2,200, 2,100) in the sensitivity analysis. The direction of effect (lower calcification in the STS group) was preserved, but statistical significance was lost owing to reduced statistical power and wider CrI. For EA, the effect estimate remained numerically comparable: −1,300 (95% CrI −4,300, 1,600) in the sensitivity analysis versus −1,330.06 (95% CrI −3,810.76, 1,145.84) in the primary analysis.

For the remaining three calcification endpoints – coronary Agatston score (online suppl. Fig. S6A), aortic Agatston score (online suppl. Fig. S6B), and aortic valve calcification (AVC; online suppl. Fig. S6C) – all interventions retained the same direction of effect relative to standard care as seen in the primary analysis, with no reversal of effect direction detected. In summary, exclusion of studies with some concerns or high risk of bias did not reverse the direction of treatment effects for any endpoint. The consistency in effect direction supports qualitative robustness of the primary trends, although statistical significance was lost for CAC volume score due to substantially reduced sample size and statistical power. This indicates that the significant effect of STS on CAC volume score is partially driven by studies judged to have some concerns of bias. This is likely attributable to the substantial loss of statistical power after removing nearly one-quarter of studies, rather than a genuine bias-driven effect.

## Discussion

To our knowledge, this is the first NMA to systematically compare the relative efficacy of seven pharmacological agents – including STS, cinacalcet, EA, SNF472, VK1, VK2, and atorvastatin – for improving VC and VS in dialysis patients, based on 12 RCTs including 979 dialysis patients. Probabilistic SUCRA rankings suggest a potential trend that STS may be associated with greater improvement in VS relative to other interventions, while EA may be associated with greater reduction in CAC volume score. However, most pairwise comparisons did not reach statistical significance, and definitive conclusions about relative superiority cannot be drawn from the current evidence. Notably, standard care ranked highest in SUCRA for coronary artery Agatston score, aortic Agatston score, and AVC, which may be attributed to small sample sizes of relevant studies leading to unstable effect estimates, high baseline variability in calcification severity among patients receiving active treatments, or potential adverse effects of drugs indirectly affecting calcification scores. This counterintuitive ranking is most likely a statistical artifact of sparse networks and wide CrI and does not indicate that active treatments are harmful or ineffective. However, standard care lacks direct anti-calcification and anti-stiffness effects, and its apparent superiority should be interpreted cautiously. The outcomes analyzed (PWV, CAC score, etc.) are surrogate markers for cardiovascular events, and while strongly associated with cardiovascular morbidity and mortality in dialysis patients, they do not directly reflect hard endpoints (e.g., myocardial infarction, stroke, all-cause mortality). Only 3 of the 12 included studies reported cardiovascular endpoints, with no significant differences between groups. Future studies should prioritize hard endpoints to confirm clinical benefit, and large-sample studies are needed to verify these findings.

Mechanistically, the anti-calcification effect of EA is primarily attributed to its direct binding to hydroxyapatite crystals and inhibition of calcium-phosphate mineral aggregation; it also reduces the release of pro-calcification factors and upregulates anti-calcification proteins such as osteopontin in vascular wall cells, indirectly suppressing calcification progression.

For STS, three core pathways are thought to underpin its dual benefits for VS and VC. First, as a reducing agent, STS forms soluble complexes with calcium ions to lower free calcium concentration and reduce calcium phosphate precipitation [9, 26, 27]. Second, it scavenges reactive oxygen species to alleviate oxidative damage to vascular smooth muscle cells and inhibit their osteogenic differentiation, which further restores endothelial dysfunction and indirectly delays calcification progression [28]. Third, its mild vasodilatory activity directly improves vascular compliance [11, 29–31]. These mechanisms have been validated in both uremic animal models and clinical HD cohorts and align with the probabilistic ranking trend of STS showing favorable effects on both stiffness and calcification outcomes in our analysis.

Safety considerations are paramount for clinical translation. STS is associated with nausea, vomiting, and metabolic acidosis. EA showed a probabilistic ranking advantage for reducing CAC volume score, making it a candidate option for patients with heavy calcification burden or rapid calcification progression when balanced against safety risks. However, long-term EA use may increase adynamic bone disease risk in some patients, so bone turnover markers (alkaline phosphatase, PTH) should be monitored, and dose adjustment or discontinuation considered if needed. Cinacalcet can cause hypocalcemia and gastrointestinal discomfort, while VK2 has a favorable safety profile. Due to limited reporting of adverse events in included studies, quantitative synthesis was not feasible, but clinicians should monitor these risks. EA should be used cautiously in patients at high risk of adynamic bone disease, and STS or cinacalcet may require dose adjustment for those with gastrointestinal disorders.

In contrast, drugs like VK1, VK2, and atorvastatin, although they have potential effects on metabolic regulation, are less effective in directly reversing VC. The limited efficacy of VK1 and VK2 may be due to the fact that VC is a long-term, progressive process, and in CKD, the occurrence and development of VC involve factors such as serum phosphate, serum calcium, PTH, and calcium levels in dialysate. These factors are interrelated through complex interactions, and the effects of vitamin K may be too short-lived to reverse existing calcific lesions [32]. As a mechanistic hypothesis proposed by previous studies, the limited efficacy of atorvastatin may be related to mismatches between its pharmacological targets and the core pathophysiology of VC in dialysis patients. Similarly, SNF472 may show limited improvement due to its modest phosphate-lowering effect and lack of direct structural effects on established vascular lesions. These interpretations are derived from existing literature and are not directly confirmed by the present analysis.

Our findings are consistent with previous studies, showing that STS can delay the progression of VC and VS in HD patients. Probabilistic rankings suggest STS as a promising dual-acting candidate that may simultaneously improve VS and slow calcification progression, but this requires further validation in larger head-to-head trials. Although EA ranks highest for CAC volume score reduction, its effect on improving vascular function is limited, suggesting that combination or sequential therapy may be a future research direction. Clinically, STS may be considered as an exploratory treatment option for dialysis patients with both VC and VS, especially for those who have poor responses to traditional phosphate-lowering treatments, but decisions should be made with full consideration of the current evidence limitations.

### Limitations

This NMA also has several potential limitations. First, although it systematically integrated RCTs of various drugs, the overall sample size of the included studies remains limited, and the follow-up periods were generally short. Most trials lasted only 6–12 months, which may not be sufficient to fully reflect the long-term effects of the drugs on chronic progressive VC and VS. Second, several outcomes (coronary Agatston score, aortic Agatston score, AVC) included only 3 studies, resulting in sparse network structures, limited statistical power, and potentially unstable treatment rankings. Conclusions for these outcomes are exploratory and should be interpreted with caution. Third, there were differences in drug doses, administration routes, and treatment durations between studies, which could introduce heterogeneity and confound direct comparisons of effect sizes. Differences in the methods used for evaluating VC (such as CT imaging parameters, scoring algorithms, or regional definitions), as well as dialysis methods (HD vs. PD), could also introduce potential biases. Notably, there is considerable interindividual variability in the baseline disease profiles, nutritional status, and calcium-phosphate metabolism levels of dialysis patients, and these factors themselves have a significant impact on the progression of calcification and stiffness. We used random-effects models to partially address heterogeneity, but residual confounding cannot be fully excluded. Fourth, for most outcomes with fewer than 10 included studies, standard publication bias tests (funnel plot, Egger’s test) have limited statistical power, and publication bias cannot be reliably excluded. Finally, this study was limited to dialysis patients, and it did not evaluate the potential effects of these drugs in kidney transplant recipients or early-stage CKD patients. Future research should include more rigorously designed, larger-sample, long-term prospective head-to-head randomized controlled trials to further validate the reliability and generalizability of these findings.

### Clinical Implications

These findings carry tentative clinical implications given the limitations of the current evidence base, and provide a comparative efficacy framework for pharmacological management of VC and VS in dialysis patients. Probabilistic SUCRA rankings suggest that STS has a potential comprehensive advantage in improving VS and slowing calcification progression. Through calcium ion chelation and antioxidant effects, STS not only slows the deposition of calcium phosphate but also improves vascular compliance, suggesting its potential value as a vascular protective strategy in dialysis patients. EA showed a probabilistic ranking advantage for reducing CAC volume score, making it a candidate option for patients with heavy calcification burden or rapid calcification progression when balanced against safety risks. The complementary mechanisms of STS and EA suggest that future studies may explore the potential value of combination or sequential therapies.

In summary, this study provides evidence-based insights to inform clinical drug selection, with STS being a reasonable exploratory option for dialysis patients with concurrent VC and VS, while EA serves as a beneficial complement for targeted anti-calcification therapy. Future research should focus on large-scale, long-term head-to-head RCTs comparing STS and EA, prioritize hard cardiovascular endpoints, standardize intervention protocols to reduce heterogeneity, and explore individualized treatment strategies based on dialysis modality and baseline calcification status.

## Conclusion

This NMA indicates that probabilistic SUCRA rankings suggest STS has a potential comprehensive advantage in improving VS (highest SUCRA = 89.4%) and delaying calcification progression (second-highest SUCRA = 76.9% for CAC volume score). STS may be a reasonable exploratory option for renal dialysis patients with concurrent VC and VS, but this finding requires further validation given the absence of statistically significant differences for several outcomes. EA ranks highest in SUCRA for reducing CAC volume score but has limited effect on VS and requires caution regarding long-term safety. Overall, current evidence remains limited by small sample sizes, sparse networks for some outcomes, and mostly nonsignificant between-group comparisons. Large-scale, head-to-head randomized controlled trials are needed to further verify these conclusions.

## Statement of Ethics

A Statement of Ethics is not applicable because this study is based exclusively on published literature.

## Conflict of Interest Statement

None of the authors had any conflicts of interest.

## Funding Sources

This study was not supported by any sponsor or funder.

## Author Contributions

conceptualization and writing – original draft preparation and supervision: Y.L.; writing – review and editing: all authors; methodology and resources: J.W. and Y.T.; visualization: J.C.; formal analysis: Y.L. and J.W.; project administration and investigation: Q.P.

## Data Availability

All data generated or analyzed during this study are included in this article and its online supplementary material. Further enquiries can be directed to the corresponding author.
